# Effect of defects controlled by preparation condition and heat treatment on the ferromagnetic properties of few-layer graphene

**DOI:** 10.1038/s41598-017-06224-w

**Published:** 2017-07-19

**Authors:** Qinghua Miao, Lidong Wang, Zhaoyuan Liu, Bing Wei, Jinhui Wang, Xiangli Liu, Weidong Fei

**Affiliations:** 1 School of Materials Science and Engineering, Harbin Institute of Technology, Harbin, 150001 China; 2 School of Mechanical Engineering, Qinghai University, Xining, 810016 China; 3 Department of Materials Science and Engineering, Shenzhen Graduated School, Harbin Institute of Technology, Shenzhen, 518055 P.R. China

## Abstract

Magnetism in graphene has stimulated extensive studies to search for novel metal-free magnetic device. In this paper, we use a synthesis method far from equilibrium state named self-propagating high temperature synthesis (SHS) to produce few-layer graphene with different defect contents and then use a heat treatment process (vacuum-annealing and air-cooling) to further control the defects in graphene. We find that the type and content of defects in graphene can be controlled by adjusting the mole ratio of reactants (Mg: CaCO_3_) for SHS reaction and the temperature of the subsequent heat treatment. The deviation of the ratio of reactants from stoichiometric ratio benefits the production of graphene with higher concentration of defects. It is indicated that the temperature of the heat treatment has remarkable influences on the structure of graphene, Raman-sensitive defects can be recovered partly by heat treatment while IR-sensitive defects are closely related with the oxidation and decomposition of the oxygen-containing groups at elevated temperature. This work indicates that SHS is a promising method to produce graphene with special magnetism, and the heat treatment is an effective way to further adjust the magnetism of graphene. This work sheds light on the study to develop carbon materials with controlled ferromagnetism.

## Introduction

Graphene has generated a lot of activity in the area of material science due to its exceptional electronic and mechanical properties^1, 2^. Compared with other properties, magnetism in graphene^3–8^ has stimulated extensive studies to search for novel metal-free magnetic device. The emergence of magnetism in versatile natured graphene and the ability to control its properties can lead graphene to be an excellent material for spintronics and other memory based device applications which promise information storing, processing and communicating at faster speed with lower energy consumption. Research on the origin of magnetism in graphene oxide^9^, graphene nanoflakes^10–12^, hydrogenated graphene^13^ and graphene nanoribbons^14^ suggested that the magnetic behavior of graphene based materials is to a large part governed by their structures. Although the mechanism of graphene magnetism is complicated, extensive theoretical and experimental studies indicated that defects^15^, disordering^4^, covalent-adsorption^16^ and magnetic edge state in graphene nanoribbons^17^ and partially hydrogenated epitaxial graphene^13^ are the potential carriers for the magnetism in graphene.

Ferromagnetism has also been observed in graphene materials prepared by different methods like thermal exfoliation of graphitic oxide, conversion of nano diamonds, arc evaporation of graphite in hydrogen and graphene oxide partially reduced by hydrazine and further completely reduced by thermal annealing, since graphene obtained by different methods has different types and quantity of defects^13^. Recently, we have developed a facile and cost-effective method named as self-propagating high temperature synthesis (SHS) to produce few-layer graphene^18^. The SHS process utilizes the heat generated by the exothermic reaction of Mg and CaCO_3_ to sustain itself in the form of a combustion wave after external ignition. The process is of high reaction temperature, fast heating and cooling speed and far from equilibrium state, so the defect in graphene made by this method is special. We have found that few-layer graphene samples both non-doped and doped with nitrogen produced by SHS method exhibit ferromagnetic properties and have high Curie temperatures (>600 K), and the saturation magnetization and coercive field increase with the increasing of nitrogen contents in the samples^19^. Taking advantage of the far-from-equilibrium-state SHS process, people are expected to produce graphene with different kinds and contents of defects, which helps further clarify the relationship between defects and the ferromagnetic properties of graphene. However, few works have been done on these issues.

In the present study, firstly, we explored the method to produce few-layer graphene with different defect concentrations by changing the ratio of reactants (Mg: CaCO_3_) in SHS process. Secondly, in order to further improve the magnetic property of SHS graphene, we proposed a heat treatment method (vacuum-annealing and air-cooling), which is heating the sample in vacuum environment at a certain high temperature and then cooling down to room temperature in atmospheric environment. Our works indicated that the deviation of stoichiometric ratio of the reactants under far from equilibrium state is a promising method to produce graphene with special magnetism, and that the designed heat treatment is an effective way to further adjust the ferromagnetism of graphene.

## Experimental

### Synthesis of graphene

Here, we used the SHS method to synthesize graphene with different content of defects by changing the ratio of reactants: magnesium, (99.5% purity) and calcium carbonate (CaCO_3_, 99.5% purity); these materials were purchased from Sinopharm Chemical Reagent Co., Ltd.

The SHS experiments were conducted in a stainless-steel combustion chamber under an atmosphere of carbon dioxide (99.9%)^19^. In order to investigate the effect of reactant composition on the chemical and ferromagnetic properties of graphene, the molar ratios of Mg and CaCO_3_ were chosen as 2:1 and 4:1; the ratio (2:1) is a stoichiometric ratio according to the reaction: 2Mg + CaCO_3_ = 2MgO + CaO + C (graphene), while the ratio (4:1) was designed to deviate from the stoichiometric ratio. The products were expressed as M2C1 and M4C1, respectively, according to the ratios of Mg and CaCO_3_. 16 grams of Mg for M2C1 and 32 grams for M4C1 were added to 33.3 grams of calcium carbonate and then milled in a mortar for 20 minutes, respectively. Each sample was ignited by an electric ignition device composed by a direct current (DC) power source and a resistance-based wire heater. The ignition current was 22 A. The coarse product was placed in dilute hydrochloric acid (10 v/v %) containing ethanol (20 wt %) and sonicated for 1 h, then washed with deionized water and absolute ethanol in that order. The obtained sample was dried in a vacuum oven at 120 °C for 24 h.

Every graphene sample (M2C1 or M4C1) was divided into 4 parts and three of them were heated at 500 K, 650 K and 800 K, and named as M2C1-500 or M4C1-500, M2C1-650 or M4C1-650 and M2C1-800 or M4C1-800, respectively. As a contrast, the initial M2C1 and M4C1 sample without heat treatment was named as M2C1-G and M4C1-G (G stands for the generated graphene). The heating rate from room temperature to the desired temperature was 5 K·min^−1^ and kept for 5 min in vacuum (10^−4^ Pa), then cooled down to room temperature within 5 minutes in air by opening the valve.

### Characterization techniques

The phase composition of the as-prepared powders was analyzed by powder X-ray diffraction (XRD) analyses (Philips X’Pert diffractometer) with CuKα radiation. Environmental scanning electron microscopy (ESEM, Helios Nanolab 600i) and high-resolution transmission electron microscopy (HRTEM JEM-2100) were used to observe the morphology of the graphene sheets. The TEM specimens were prepared by dropping ethanol/water (38 v/v %) solution containing 1 wt % graphene onto a copper grid and drying at 100 °C. Raman spectra was obtained using a Raman Station (B & WTEK, BWS435-532SY) with a 532 nm wavelength laser corresponding to 2.34 eV. X-ray photoelectron spectroscopy (XPS, Thermo Fisher) was utilized to determine the bonding characteristics of the samples. All XPS peaks were calibrated according to the C 1 s peak (284.6 eV). The magnetic properties were measured using a Quantum Design MPMS magnetometer based on a superconducting quantum interference device (SQUID). Thermogravimetric analysis (TGA) was performed on a Netzsch STA 449 F3 under a heating rate of 10 K·min^−1^ in air atmosphere form 300 K to 1200 K. The nitrogen adsorption/desorption measurements were carried out on Belsorp mini II (Japan) at 77 K to obtain the specific surface area of M2C1-G and M4C1-G. Before adsorption/desorption tests, the samples were degassed at 150 °C for 4 hours with vacuum pumping.

## Result and Discussion

**Figure 1:**
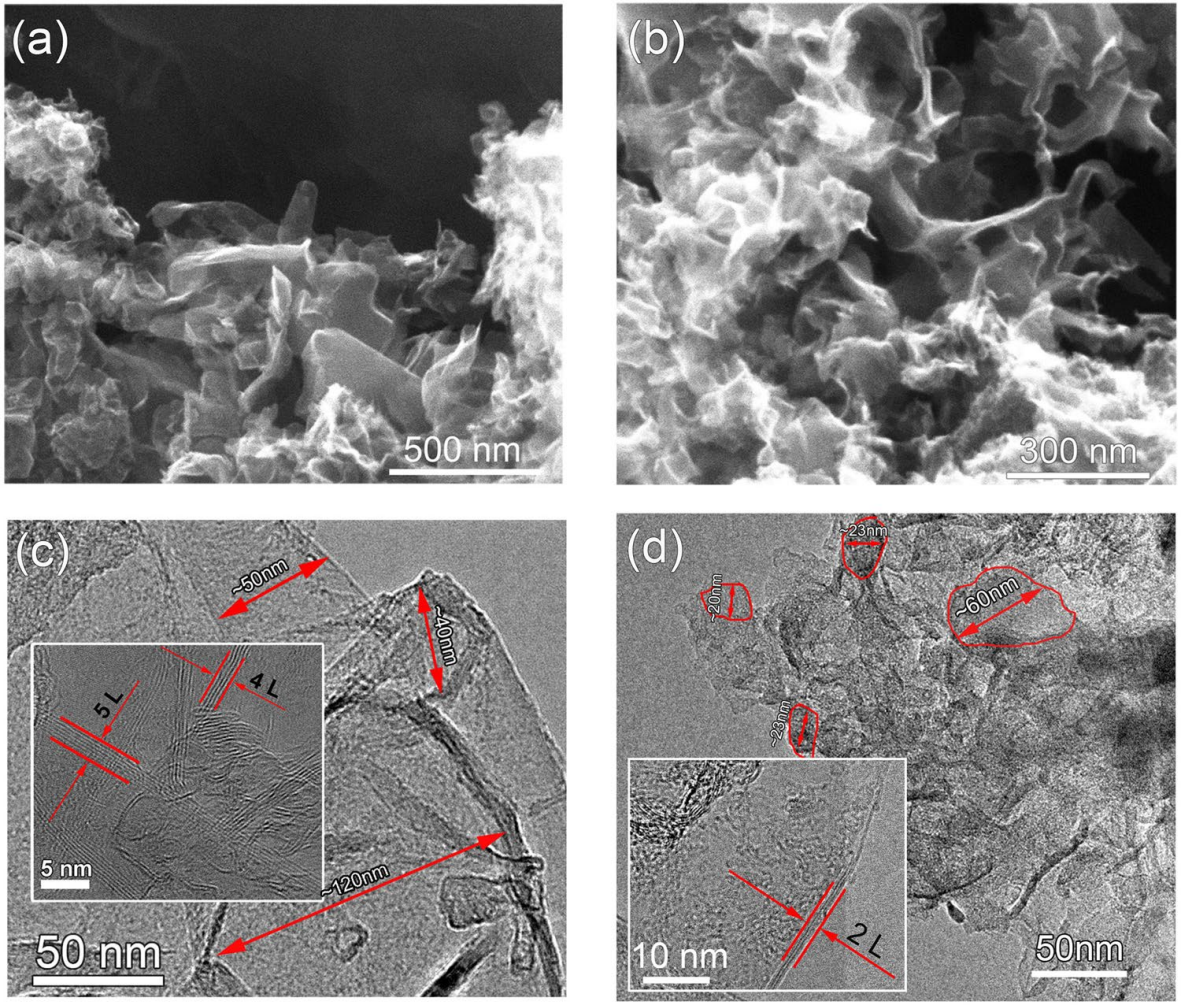
The morphologies of few-layer graphene: (**a**) and (**b**) SEM of M2C1-G and M4C1-G, (**c**) and (**d**) TEM of M2C1-G and M4C1-G.

Figure 1 shows the typical SEM and TEM images of the SHS products. Figure 1(a) and (b) are the SEM images of M2C1-G and M4C1-G, respectively. In the images, thin corrugated sheets can be found assembled together, showing a three dimensional porous structure. In addition, the EDX of both samples have been provided in the Supplementary Information Fig. S1 and Table S1. It reveals that both M2C1-G and M4C1-G are mainly composed of C and O, and a small amount of Ca and Mg. The components of their composition have been list in Table S1. In the sample of M4C1-G, the contents of magnesium and calcium are less than those in M2C1-G, which are consistent with the results of XPS. Figure 1(c) and (d) show the TEM images of M2C1-G and M4C1-G, respectively. The typical diameters of flakes have been marked as shown in the Fig. 1(c) and (d). The diameters of flakes in M2C1-G are in the range of 40–120 nm, while those in M4C1-G are in the range of 20–60 nm, which reveals that the diameters for M2C1-G sheets are mostly larger than those for M4C1-G sheets. In addition, the number of the layers of these edges in Fig. 1(c) approximately ranges 2–10, while that for M4C1-G ranges 2–4 which are obviously thinner as shown in the inset of Fig. 1(d). The adjacent layer spacing in the insets of Fig. 1(c) and (d) thickness is about 0.34 nm which is the characteristic of few-layer graphene. The specific surface area (SSA) of the generated graphene was investigated by Brunauer–Emmmett–Teller (BET) measurement. The SSA of M2C1-G is up to 358 m^2^ g^−1^ according to the BET method. Then we can evaluate the layer of M2C1-G is about 7 through the SSA of monolayer graphene is 2630 m^2^ g^−1^. According to the above analysis and the Raman analyses in the following section, we conclude that the products synthesized by SHS method are few-layers graphene.

The difference of the morphology between M2C1-G and M4C1-G can be understood by considering the mole ratios of reactants (Mg: CaCO_3_) for SHS reaction. The stoichiometric mole ratio of the reaction between Mg and CaCO_3_ is 2:1, which is just the ratio for M2C1-G, while the ratio for M4C1-G is 4:1, much higher than the stoichiometric ratio. The deviation of the stoichiometric ratio for M4C1 means that Mg is excessive for the reaction, and the excessive Mg may play multiple roles in the SHS reaction. Firstly, the excessive Mg may melt at 648 °C and volatilize at 1107 °C, which can absorb large amount of heat produced by the exothermic SHS reaction (ΔH = −632 kJ/mol) and decrease the maximum temperature of the reaction. Secondly, the gaseous Mg in the enclosed space of reaction container may affect the growth process of graphene since they may decrease the collision probability of the reactive carbon atoms produced during the SHS reaction process. As a result, we can deduce that the reaction temperature for M4C1-G is lower than that of M2C1-G which benefits the production of smaller and thinner sheets for M4C1-G as shown in Fig. 1. Of course, this is only the basic discussion on the phenomenon, to further understand the roles of the excessive Mg, more work should be done to clarify the mechanism of the SHS reaction.

**Figure 2:**
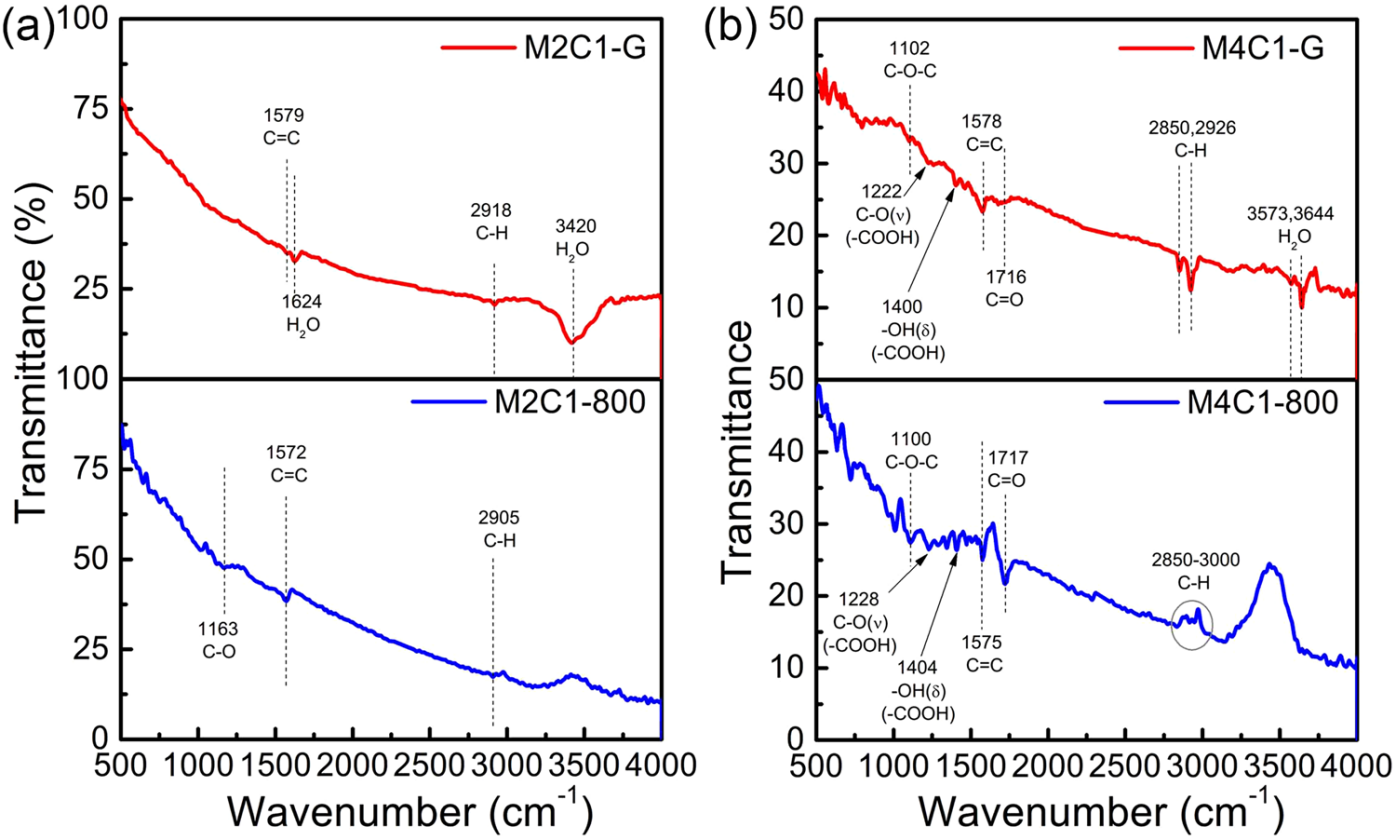
FTIR spectra of M2C1 (**a**) and M4C1 (**b**) at the heat treated temperature of 300 K and 800 K.

Figure 2 shows the FTIR spectra of M2C1-G, −800 and M4C1-G, −800. The absorption peak around 1575 cm^−1^ is ascribed to the skeletal vibration of aromatic ring (C=C stretching vibration); the peaks at 1141 cm^−1^, 1717 cm^−1^, 2850–2920 cm^−1^ and 3200–3600 cm^−1^ are attributed to the C-O-C, C=O, C-H and O-H vibration, respectively. On the one hand, from Fig. 2(a) it can be found that the peaks corresponding to H_2_O (1624 cm^−1^) and O-H vibration (3200–3600 cm^−1^) with the increase of heat treatment temperature, suggesting the remove of hydroxyl and water on graphene; the peaks corresponding to oxygen-containing groups are not clear for M2C1-G, suggesting that M2C1-G has good chemical stability. On the other hand, it is interesting to see that the FTIR spectra of M4C1-G is quite different from that of M2C1-G. The peaks corresponding to epoxy, hydroxyl, carbonyl and carboxyl groups can be both found for M4C1-G and M4C1-800; however, the relative intensities of peaks corresponding to epoxy, carbonyl and carboxyl groups for M4C1-800 increase obviously, while the peaks corresponding to O-H vibration almost disappear, compared with those for M4C1-G. Consequently, it can be concluded that M2C1-G has less oxygen-containing groups and is more stable for the heat treatment than M4C1-G and that the oxidization of graphene happens for M4C1-G heat-treated at high temperature.

To better study this behavior, we performed the Thermogravimetric Analysis (TGA) and the differential scanning calorimetry (DSC) of M2C1-G and M4C1-G in air environment at the heating rate of 10 K·min^−1^ and the result has been added in Fig. S2 (Supporting Information). From the curves of DSC, two exothermic peaks can be seen, corresponding to the two weight loss stages from the curves of TG. The first exothermic peak is located at 776 K for M2C1-G and 740 K for M4C1-G, while the second exothermic peak is at 906 K and 884 K for M2C1-G and M4C1-G, respectively. The first exothermic peak is small compared with the second one for M2C1-G, while they are almost equal for M4C1-G. Accordingly, there are two weight loss steps for the SHS graphene. The first step of weight loss occurs at the temperature range of 300–830 K for M2C1-G and 300–670 K for M4C1-G, corresponding to the removal of adsorbed water and the labile oxygen-containing groups. The second mass loss range is from 830 K to 950 K for M2C1-G and 670 K to 950 K for M4C1-G, which is assigned to the combustion of the carbon skeleton of graphene, releasing CO and CO_2_. From the results of DSC and TG analysis, we conclude that the SHS graphene had two types of structure. One is easily oxidized at low temperature, corresponding to the oxygen-containing groups and carbon defects; the other is more thermally stable, oxidized at higher temperature, assigning to the defect-free parts in SHS graphene^20^. But the ratio of the two exothermic peaks in the curves of DSC for the two samples is different. The relatively intensity of the first peak to the second peak in M4C1-G is much higher than that in M2C1-G, indicating that M4C1-G contained more oxygen-containing groups and its thermal stability is lower than that of M2C1-G, which are consistent with the results from FTIR and XPS.

**Figure 3:**
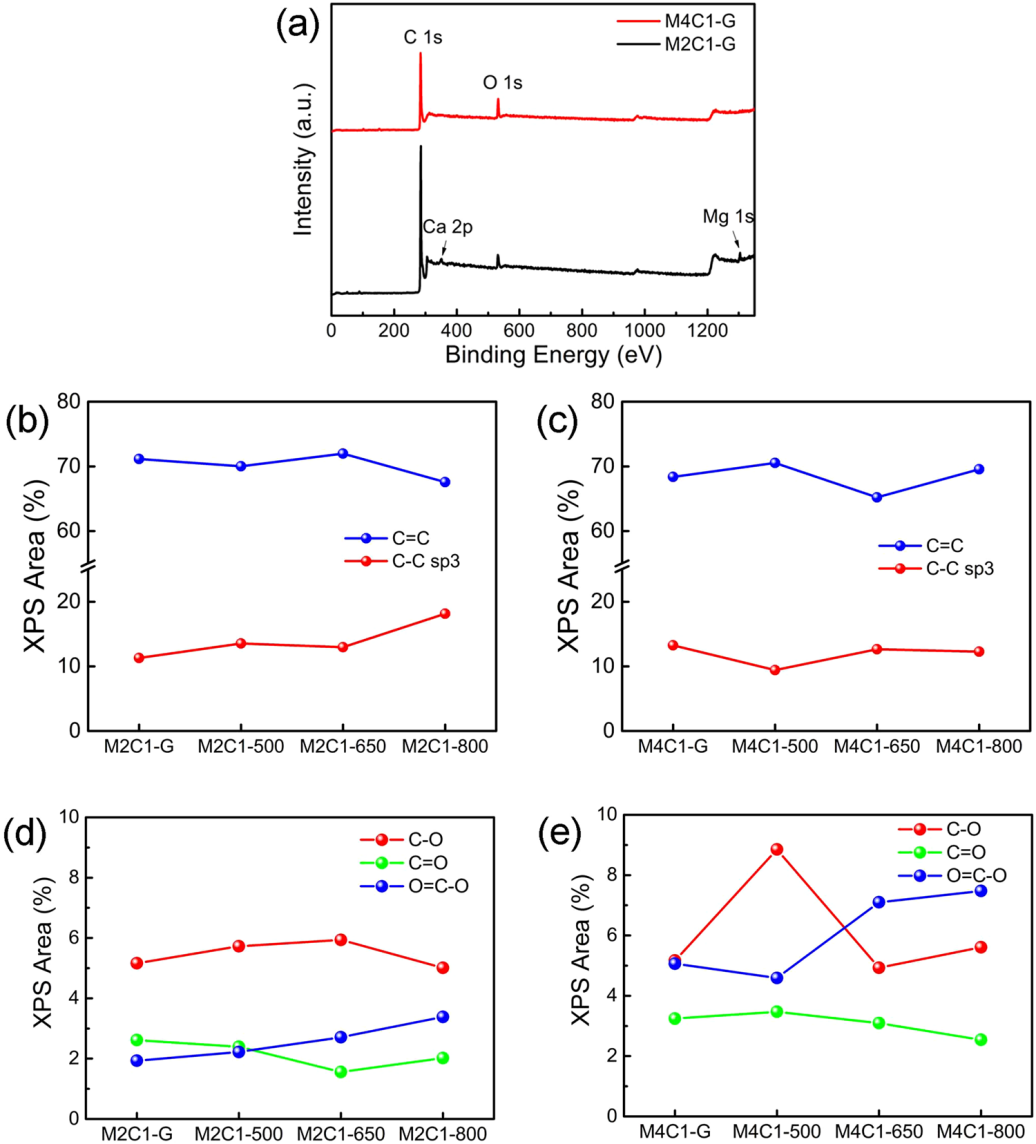
(**a**) XPS survey spectra of M2C1-G and M4C1-G; (**b**–**e)** The compositions of components from C 1 s in M2C1 ((**b**) and (**d**)) and M4C1 ((**c**) and (**e**)).

XPS characterizations are further performed to analyze the elemental composition and C/O configuration in the samples. The XPS survey spectra of the samples in Fig. 3(a) show the presence of carbon, oxygen, magnesium and calcium elements, which is in agreement with the result of XRD. The high resolution C 1 s spectra of M2C1 and M4C1 heat-treated at different temperatures are shown in Fig. S3 and S4, respectively. The spectra are analyzed by XPSpeak41 software and corrected for the background signals using the Shirley algorithm prior to curve resolution^21^. Gaussian decomposition and Lorentz decomposition are employed in this fitting. The C1s component can be deconvolved into six components: sp^2^ C=C (284.4 ± 0.1 eV), sp^3^ C-C (285.4 ± 0.1 eV), C-O (286.4 ± 0.1 eV), C=O (287.5 ± 0.2 eV), O=C-O (288.6 ± 0.2 eV) and π-π* satellite peak (290.5 ± 0.1 eV)^22–24^. In order to obtain more detailed information, the contents of components in C 1 s of M2C1 and M4C1 treated at different temperatures are analyzed according to the fitting and the results are shown in the Fig. 3(b,c,d and e).

Figure 3(b) and (c) demonstrate the effect of heat treatment temperature on the XPS areas for C=C and C-C bonds. For M2C1 sample, the content of XPS area for C=C has a small fluctuation in the treatment temperature range 300 to 650 K and then decreases for 800 K. Interestingly, it is clear to find that the content of XPS area for C-C has an opposite trend. Since C=C and C-C bonds are related with sp^2^ and sp^3^ C in graphene, the well opposite trend suggests that the oxidized sp^2^ carbons are mostly changed to sp^3^ carbons and vice versa. Similar trend can also be found in M4C1 sample.

XPS results in Fig. 3(d and e) give us information about the effect of heat treatment temperature on the contents of oxygen functional groups. Firstly, it can be found that the contents of carboxyl group in both M2C1 and M4C1 have an increasing trend with the increase of the heat-treatment temperature. The content increase (2.4%) of carboxyl group from M4C1-G to M4C1-800 is higher than that (1.45%) of M2C1 samples. Secondly, for the content of C-O group, it changes relatively small for M2C1 with the increase of heat treatment temperature but fluctuates largely for M4C1 heat-treated at 500 K, suggesting that M4C1 is easier to be oxidized at 500 K to produce C-O group (hydroxyl or epoxy group) and then the group decomposed at higher temperature. Thirdly, the contents of C=O and their fluctuation for M2C1 and M4C1 are relatively small. As a result, the content of groups in M2C1 is relative stable compared with those in M4C1, the results also give us valuable information for the explanation of the ferromagnetic properties of SHS graphene.

**Figure 4:**
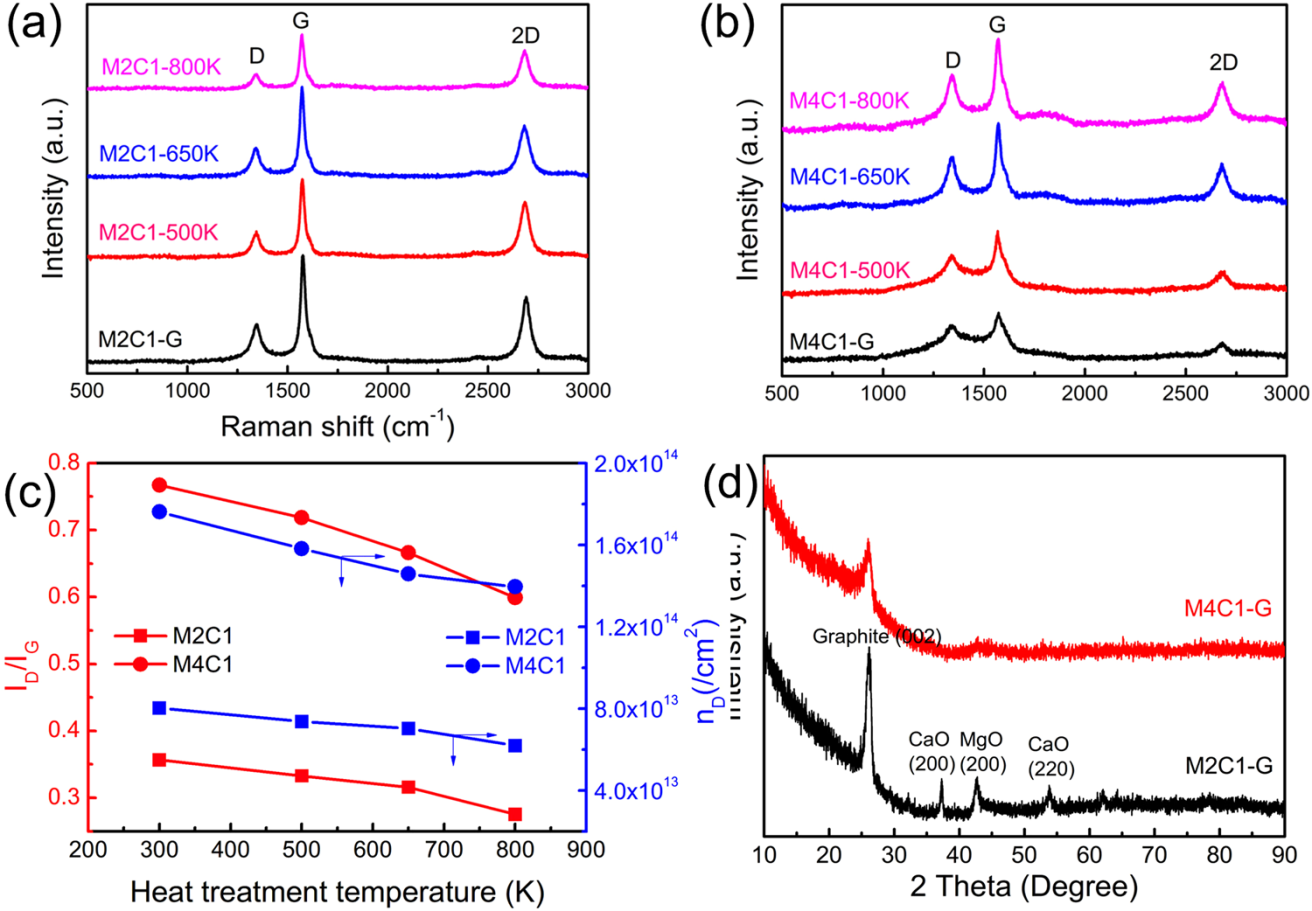
Raman spectra of M2C1 (**a**) and M4C1 (**b**) and the intensity ratios (*I*_D_/*I*_G_) (red) and the defect concentration n_D_ (blue) of M2C1 and M4C1 corresponding to the heat treatment temperatures (**c**). XRD patterns of M2C1-G and M4C1-G at room temperature (**d**).

Raman spectroscopy is considered to be an effective tool for characterization of mono-, few-, or multil-layer graphene^25–28^. The Raman spectra of the M2C1 and M4C1 samples treated at different temperatures are shown in Fig. 4(a and b). The Raman spectra of M2C1 and M4C1 show three peaks. The G band at 1570 cm^−1^ represents the in-plane bond-stretching motion of the pairs of sp^2^ hybridized C atoms (the E_2g_ phonons); the D band at 1341 cm^−1^ corresponds to breathing mode of rings or K-point phonons of A_1g_ symmetry; and the second-order D (2D) band at 2678 cm^−1^ originates from a two phonon double resonance process^28, 29^. The 2D peaks of the M2C1 and M4C1 samples around 2678 cm^−1^,which shift greatly to lower wavenumber compared with that of graphite (2714 cm^−1^), can identify the samples as few-layer graphene^30, 31^.

The relative intensity of the D peak (*I*_D_) to the G peak (*I*_G_) in graphene is directly proportional to the level of defects in the sample^32, 33^. Here, the intensity ratios (*I*_D_/*I*_G_) is used to demonstrate the relatively change of *I*_D_ to *I*_G_ with the heat treatment temperatures as shown in Fig. 4(c). The defect density (cm^−2^) of graphene can be investigated and defined as n_D_ = 5.9 * 10^14^ * E_L_^−4^ * (*I*_D_/*I*_G_)^−1^ ^29^, where the laser energy E_L_ = 2.34 eV (λ = 532 nm) and the calculated defect density is shown in Fig. 4(c), which is in the same order as that of the annealed graphene prepared by CVD reported by Park^34^ and two orders higher than highly ordered pyrolytic graphite irradiated by 140 eV Ar^+^ ions reported by Ugeda^35^.

From Fig. 4(c), it can also be found that the intensity ratio of *I*_D_/*I*_G_ of the M4C1 sample is much higher than that of the M2C1 sample treated at the same temperature. This result indicates that the mole ratio of the reactants (Mg: CaCO_3_) plays an important role on the defect density in the SHS products, the deviation of the ratio of reactants (Mg: CaCO_3_) from stoichiometric one in the case of M4C1 benefits the production of graphene with higher defect density. The ratios of *I*_D_/*I*_G_ of both M4C1 and M2C1 decrease with the increase of heat treatment temperature, while the decline for M4C1 is more obvious than that of M2C1. But the defect concentration in M4C1 is still higher than that in M2C1 overall even after heat-treated at 800 K. The reduction of defect density with the increase of the heat treatment temperature suggests the repair of the Raman-sensitive defects is the main process during the heat-treatment process. Finally, we can conclude that the ratio of reactants is the main factor for the formation of defects in SHS graphene and that the heat treatment can repair part of the defects characterized by Raman spectra, especially for M4C1.

The ratio of *I*_2D_/*I*_G_ has been used for the identification of the number of graphene layers. The value of *I*_2D_/*I*_G_ obtained from Fig. 4(a and b) for the samples treated by different temperature are shown in Fig. S5(a). It can be found that the *I*_2D_/*I*_G_ value is about 0.6 for M2C1 and about 0.4 for M4C1, which are larger than that of graphite (about 0.3)^36^. These results also indicate that M2C1 and M4C1 are few layer graphene. In addition, comparing the ratios of the samples treated by different temperatures, we can find that the ratios of *I*_2D_/*I*_G_ for M2C1 and M4C1 at any treated temperatures do not change significantly, which indicates that the heating treatment has no obvious effect on the number of graphene layers. At last, we summarize the full width at half-maximum (FWHM) of the 2D band in the spectra for all samples in Fig. S5(b). We can find that there is no obvious variation on the FWHM of the 2D band for M4C1 and M2C1 at any heating treatment temperature.

As mentioned in the research of J. T. L. Thong^37^, the FWHM of the 2D band in graphene could be a quantitative guide to distinguish the layer number (single- to five-layers) of few- layer graphene. However, it is based on graphene produced by mechanical exfoliation which has less defects, and the research about the relationship between defective graphene and FWHM of 2D peak has not yet been reported. In addition, the size of laser light spot for Raman spectra we used is about 100 μm and laser light may penetrate many graphene sheets in its light path, so the 2D peak we got reflects the information of many graphene sheets, which is composed of many overlaid 2D peaks. So the FWHM of 2D peak may not provide us with exact information about the number of SHS graphene layer.

Powder X-ray diffraction is used to analyze the phases in M2C1 and M4C1 samples as shown in Fig. 4(d). It can be found that the most intense peaks in the two XRD spectra are the peaks near 26.0° corresponding to the (002) plane of graphite. The XRD spectra are similar with the FLG in refs 30, 36. The peaks belonging to CaO (JCPDS No. 48–1467) and MgO (JCPDS No. 45–0946) can also be found for M2C1, which are the by-products of SHS reaction.

**Figure 5:**
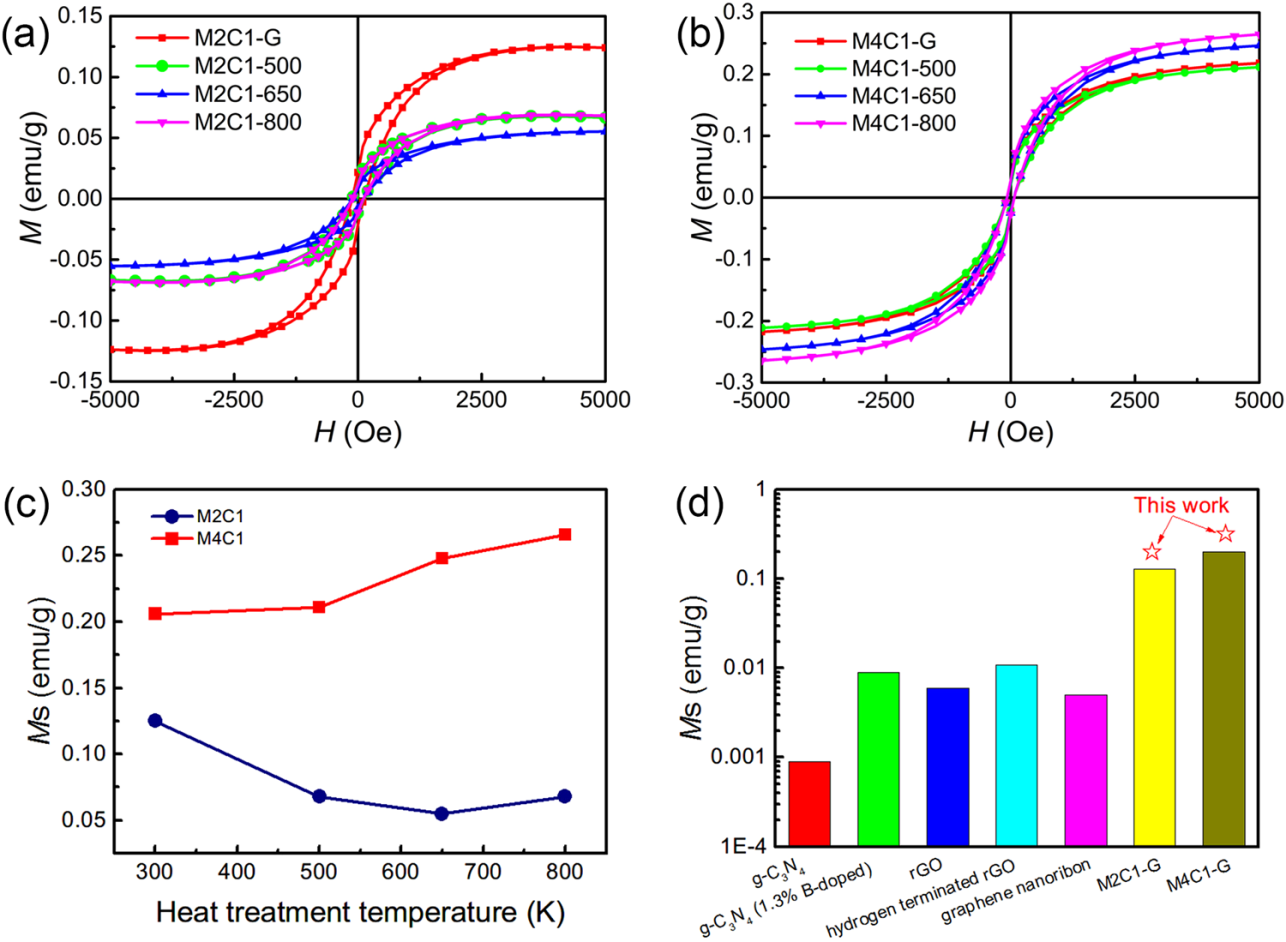
*M* versus *H* curves of M2C1 (**a**) and M4C1 (**b**); (**c**) *M*_s_ of M2C1 and M4C1 heat-treated at different temperatures; (**d**) The comparison of the values of Ms of different carbon materials at room temperature (g-C_3_N_4_^38^, g-C_3_N_4_(1.3% B-doped)^38^, rGO^39^, hydrogen terminated rGO^40^, graphene nanoribon^15^, M2C1-G and M4C1-G).

To investigate the magnetic properties of the SHS samples, the magnetization behaviors versus magnetic field curves for M2C1-G and M4C1-G and the heat-treated samples by 500 K, 650 K and 800 K are measured at room temperature (300 K) in the magnetic field range from −5000 Oe to 5000 Oe, as shown in Fig. 5(a and b). Ferromagnetism is shown clearly for M2C1 and M4C1 samples according to the magnetic hysteresis loops. The relationship between saturation magnetizations (*M*_s_) and heat treatment temperatures obtained from Fig. 5(a and b) has also been shown in Fig. 5(c). As mentioned in our former work^19^, the total ferromagnetic impurities (such as Fe, Co and Ni) in SHS graphene are less than 15 ppm, indicating the ferromagnetic contribution of impurities could be neglectable. Therefore, the results represent that the ferromagnetism of SHS graphene is due to the structure defects in it. For comparison, we further summarized the *M*_s_ values of several carbon materials at room temperature reported in the references as shown in Fig. 5(d). Among these materials, the saturation magnetization of M4C1 produced by SHS method in this paper is the highest at room temperature.

Surprisingly, it can be also found that the *M*_s_ changing tendencies of M2C1 and M4C1 heat-treated at different temperatures are quite different as shown in Fig. 5(c). Since it generally believed that the ferromagnetism is associated with defects in graphene, it is reasonable to deduce that the changing tendencies of the *M*_s_ for M2C1 and M4C1 are affected by the changes of the defects in them. The changing tendency of defect concentration for M2C1 in Fig. 4(c) is consistent with that of the *M*_s_ for M2C1, however, it is quite different for M4C1. It may indicate that Raman-sensitive defects must not be the only representation for the ferromagnetism in graphene.

It is well known that Raman measurement is sensitive to symmetric structures while FTIR spectra is sensitive to asymmetric structures. We divide the defects of the SHS graphene into Raman-sensitive and FTIR-sensitive defects. Edges (zigzag and armchair), vacancies (including single vacancy, hydrogen partially saturated vacancy and vacancy cluster) and disordering are the defects originating from the broken of the C-C bonds which make graphene sheets distorted. These defects can be measured by Raman spectrum and mentioned as Raman-sensitive. The defects corresponding to the oxygen-containing groups include carboxyl group, carbonyl group and hydroxyl group, etc., which are connected to the graphene layers by covalent bonds and also introduce various edges and defect sites. They are FTIR sensitive and mentioned as FTIR-sensitive.

We could explain the difference of the *M*_s_ tendency between M2C1 and M4C1 by considering the changes of both Raman- and FTIR-sensitive defects. On the one hand, the Raman-sensitive defects in graphene have been repaired in a certain extent as shown in Fig. 4(c), which could reduce the *M*_s_ of the SHS graphene. On the other hand, the content of carboxyl group increases with the increase of heat treatment temperature, which could increase the *M*_s_. M2C1 is more stable at elevated temperature and has less content of carboxyl and other oxygen-containing groups, so the Raman-sensitive defects play a more important role on the ferromagnetism than FTIR-sensitive one, so the *M*_s_ for M2C1 has a similar trend with the change of Raman-sensitive defects. Since M4C1 is more thermal sensitive and easy to be oxidized at higher temperature as above mentioned the effect of FTIR-sensitive defects on the ferromagnetism may overcome that of Raman-sensitive defects, as a result the *M*_s_ for M4C1 has a similar trend with the FTIR-sensitive defects. In addition, according to XPS results in Fig. 3(b–d), only the XPS area of carboxyl group has the same changing trend with that of *M*_s_ for M4C1 or M2C1, the carboxyl group must be the origin of the ferromagnetism for SHS graphene. Based on the analyses above-mentioned, we obtain a more comprehensive scene of the magnetism properties of the SHS graphene and the effect of heat treatment on them. Both Raman-sensitive and FTIR-sensitive defects could contribute to the ferromagnetic properties of graphene. The heat treatment plays an important role on elucidating the effect of different factors for the ferromagnetism of graphene.

## Conclusions

In this study, we obtain a more comprehensive scene of the ferromagnetic properties of the SHS graphene and the methods to tune them. Firstly, the deviation of the mole ratio of SHS reactants (Mg: CaCO_3_) from stoichiometric ratio benefits the production of few-layer graphene with smaller and more plicated sheets, and also benefits the production of both Raman-sensitive and FTIR-sensitive defects. Secondly, the heat treatment method could adjust the contents and types of defects by using the competitive relationship of the repair and oxidation processes. Thirdly, there are two origins of the ferromagnetism of the SHS graphene, associated with the Raman-sensitive and IR-sensitive defects respectively. The *M*_*s*_ trends could be explained by considering the changes of both Raman-sensitive and FTIR-sensitive defects. As a result, this work sheds light on the study to develop carbon materials with controlled ferromagnetism.

## Electronic supplementary material

Supplementary Information
