# Supplementary material for: Effect of defects controlled by preparation condition and heat treatment on the ferromagnetic properties of few-layer graphene
Source: Sci Rep. 2017 Jul 19;7:5877. doi: 10.1038/s41598-017-06224-w (PMC5517599; doi:10.1038/s41598-017-06224-w)
Supplement: Supplementary file 1 — Supplementary Information [file 41598_2017_6224_MOESM1_ESM.pdf]

Supplementary Information

Effect of defects controlled by preparation condition and heat treatment on the ferromagnetic properties of few-layer graphene

Qinghua, Miao [a, 1], Lidong Wang [a, 1*], Zhaoyuan Liu [a, 1], Bing Wei [a], Jinhui Wang [b*], Xiangli Liu [c*]& Weidong Fei [a,b]

a School of Materials Science and Engineering, Harbin Institute of Technology, Harbin 150001, China.

b School of Mechanical Engineering, Qinghai University, Xining 810016, China.

c Department of Materials Science and Engineering, Shenzhen Graduated School, Harbin Institute of Technology, Shenzhen 518055, P.R. China.

* Corresponding author

1. Author Qinghua Miao, Lidong Wang and Zhaoyuan Liu contributed equally to this work.

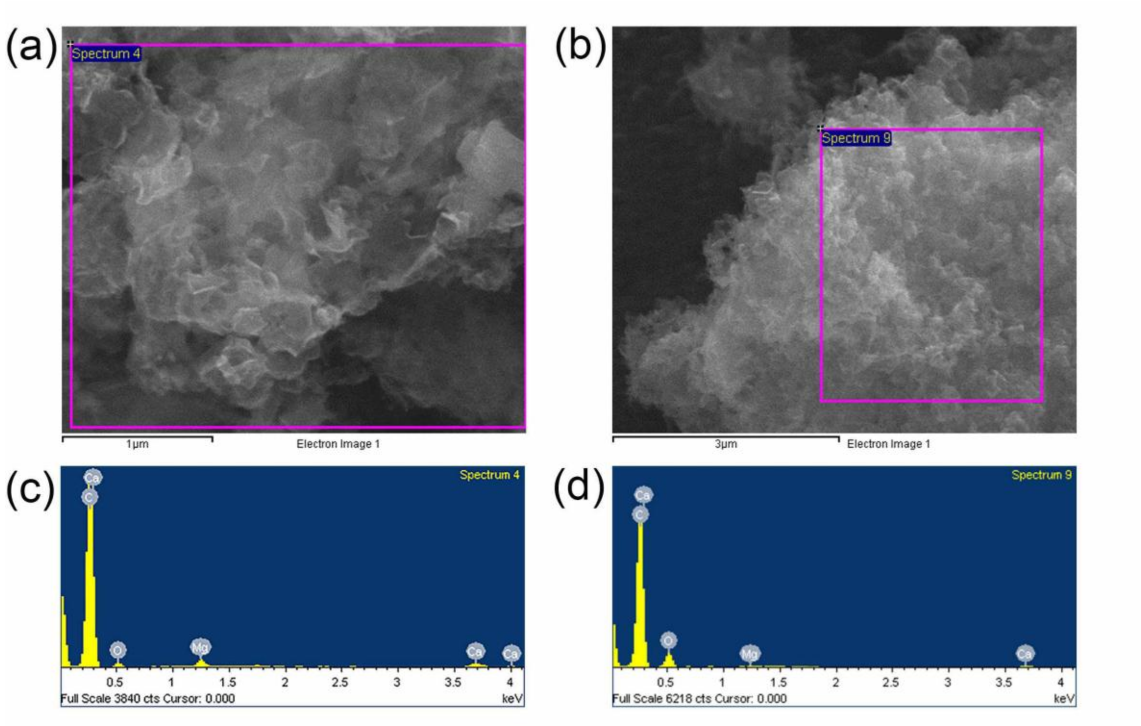

Fig. S1 SEM of M2C1-G (a) and M4C1-G (b), EDX of M2C1-G (c) and M4C1-G (d)

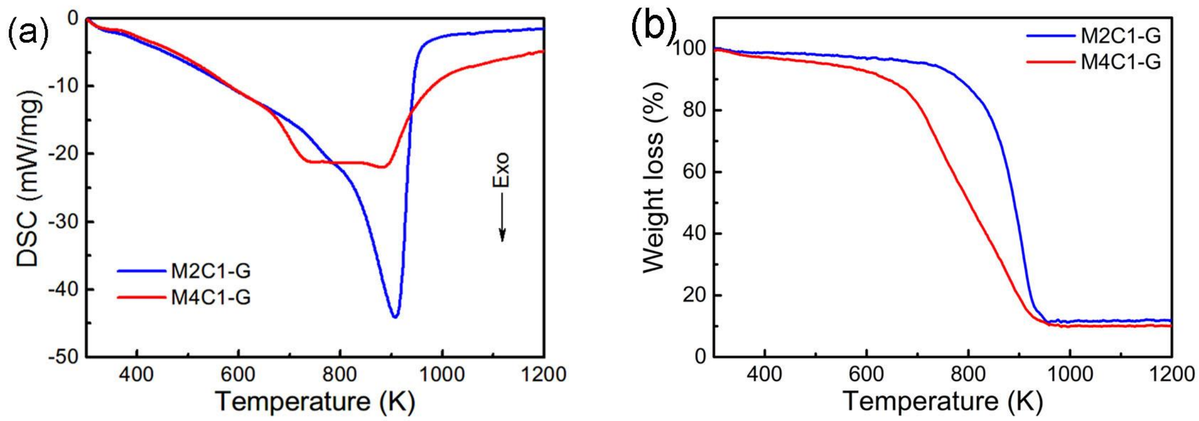

Fig. S2 TG of M2C1-G (a) and M4C1-G (b)

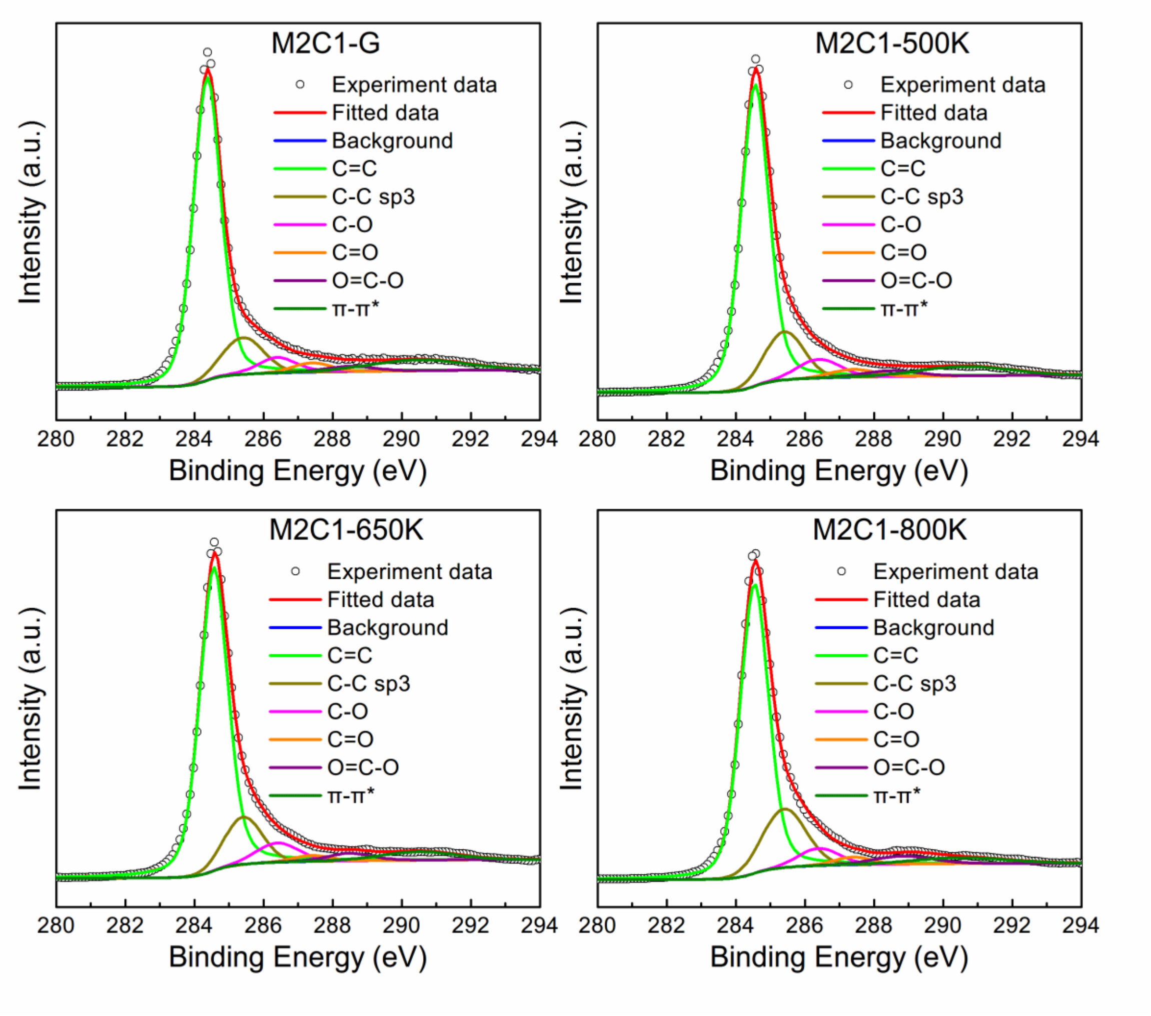

Fig. S3 XPS C 1s spectra of M2C1at different heat treatment temperatures

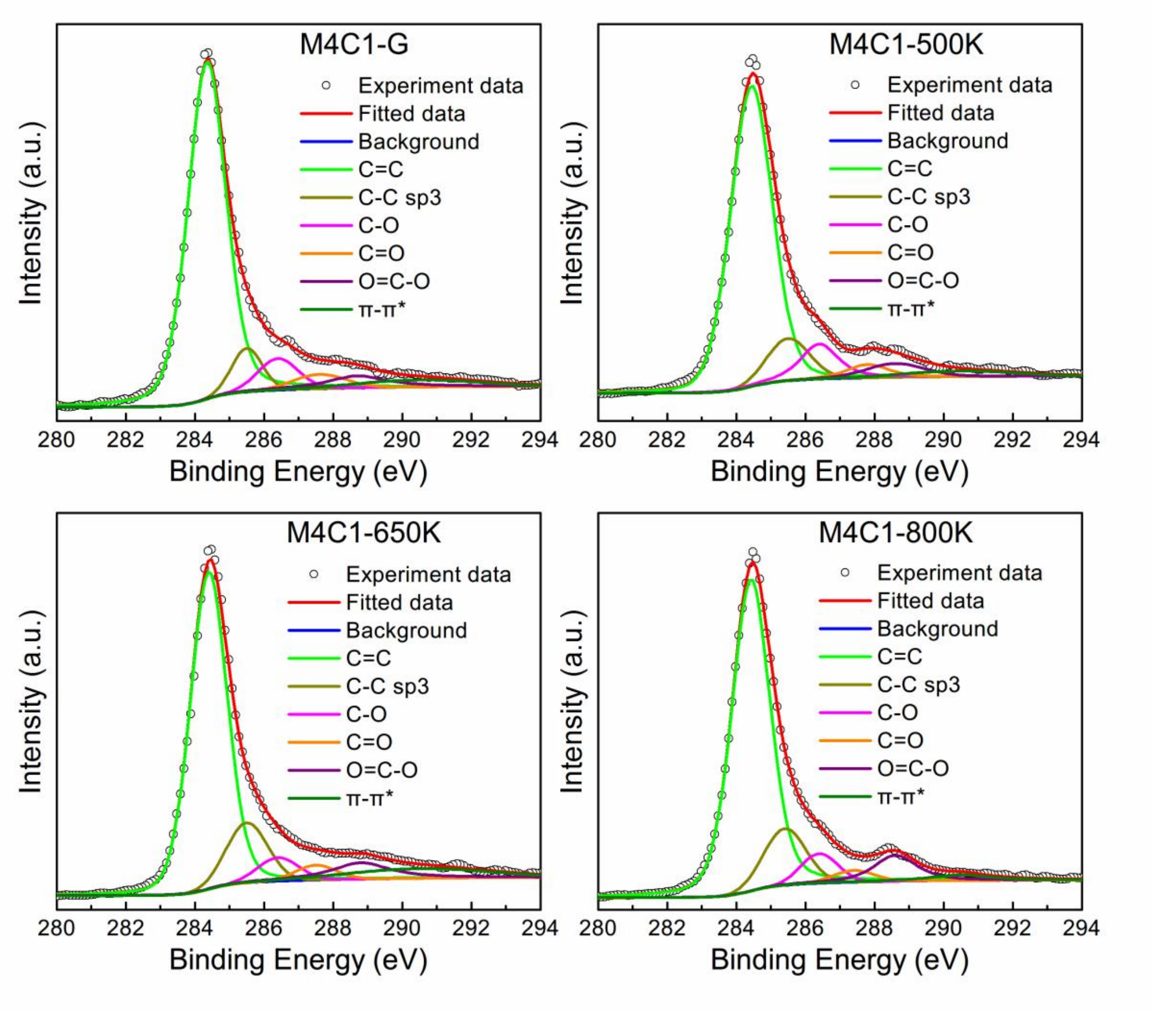

Fig. S4 XPS C 1s spectra of M4C1 at different heat treatment temperatures

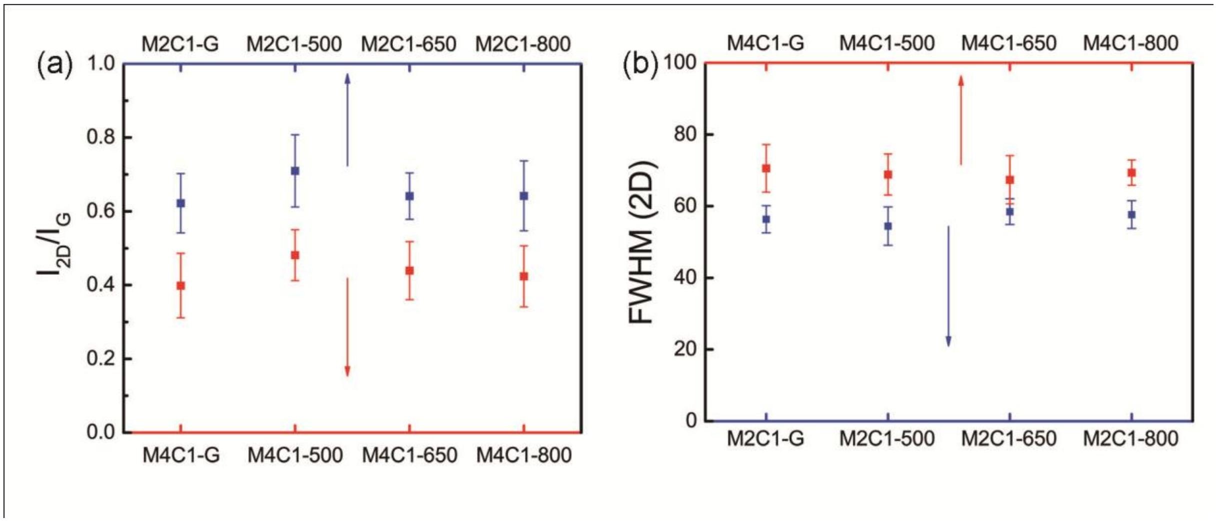

Fig. S5 The ratio of I_{2D}/I_{G} (a) and the FWHM of 2D peak (b) for M2C1 and M4C1 at different treated temperature
